# Diagnosis and management of mineral and bone disorders in infants with CKD: clinical practice points from the ESPN CKD-MBD and Dialysis working groups and the Pediatric Renal Nutrition Taskforce

**DOI:** 10.1007/s00467-022-05825-6

**Published:** 2023-02-14

**Authors:** Justine Bacchetta, Claus Peter Schmitt, Sevcan A. Bakkaloglu, Shelley Cleghorn, Maren Leifheit-Nestler, Agnieszka Prytula, Bruno Ranchin, Anne Schön, Stella Stabouli, Johan Van de Walle, Enrico Vidal, Dieter Haffner, Rukshana Shroff

**Affiliations:** 1 Reference Center for Rare Renal Diseases, Reference Center for Rare Diseases of Calcium and Phosphate Metabolism, Pediatric Nephrology Rheumatology and Dermatology Unit, Hopital Femme Mère Enfant, Boulevard Pinel, 69677 Bron, France; 2 INSERM 1033 Research Unit, Lyon, France; 3 Lyon Est Medical School, Université Claude Bernard, Lyon 1, Lyon, France; 4 Center for Pediatric and Adolescent Medicine, Im Neuenheimer Feld 430, 69120 Heidelberg, Germany; 5 Department of Pediatric Nephrology, School of Medicine, Gazi University, Ankara, Turkey; 6 Renal Unit, UCL Great Ormond Street Hospital and Institute of Child Health, London, UK; 7 Department of Pediatric Kidney, Liver and Metabolic Diseases, Hannover Medical School, Pediatric Research Center, Hannover, Germany; 8 Department of Pediatric Nephrology and Rheumatology, Ghent University Hospital, Ghent, Belgium; 9 1st Department of Pediatrics, School of Medicine, Faculty of Health Sciences, Aristotle University Thessaloniki, Hippokratio Hospital, Thessaloniki, Greece; 10 Pediatric Nephrology Unit, University-Hospital of Padova, Padua, Italy; 11 Department of Medicine (DAME), University of Udine, Udine, Italy

**Keywords:** Infants, Dialysis, Mineral and bone disorders, Calcium, Phosphate, PTH, Bone, Clinical practice points

## Abstract

**Background:**

Infants with chronic kidney disease (CKD) form a vulnerable population who are highly prone to mineral and bone disorders (MBD) including biochemical abnormalities, growth retardation, bone deformities, and fractures. We present a position paper on the diagnosis and management of CKD-MBD in infants based on available evidence and the opinion of experts from the European Society for Paediatric Nephrology (ESPN) CKD-MBD and Dialysis working groups and the Pediatric Renal Nutrition Taskforce.

**Methods:**

PICO (Patient, Intervention, Comparator, Outcomes) questions were generated, and relevant literature searches performed covering a population of infants below 2 years of age with CKD stages 2–5 or on dialysis. Clinical practice points (CPPs) were developed and leveled using the American Academy of Pediatrics grading matrix. A Delphi consensus approach was followed.

**Results:**

We present 34 CPPs for diagnosis and management of CKD-MBD in infants, including dietary control of calcium and phosphate, and medications to prevent and treat CKD-MBD (native and active vitamin D, calcium supplementation, phosphate binders).

**Conclusion:**

As there are few high-quality studies in this field, the strength of most statements is weak to moderate, and may need to be adapted to individual patient needs by the treating physician. Research recommendations to study key outcome measures in this unique population are suggested.

**Graphical Abstract:**

**Figure:**
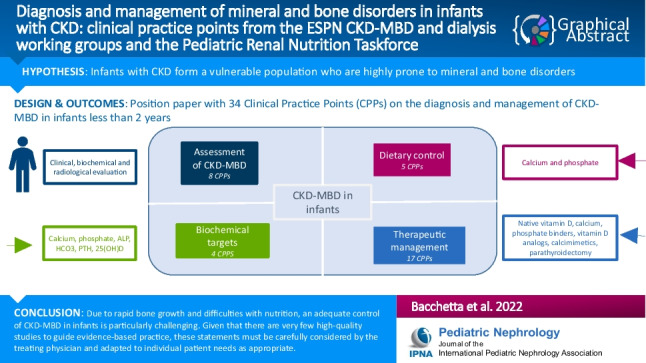
A higher resolution version of the Graphical abstract is available as Supplementary information

**Supplementary Information:**

The online version contains supplementary material available at 10.1007/s00467-022-05825-6.

## Introduction

Chronic kidney disease (CKD) affects 1 in 10,000 live births. Although survival of infants requiring chronic dialysis has improved over the past years, both morbidities and mortality remain higher in the youngest patients [1–5]. Infants with CKD are especially prone to mineral and bone disorders (MBD). Infancy represents the period of most rapid growth resulting in high demands of calcium (Ca) and phosphate (P) in order to build a positive mineral balance and adequate endochondral ossification [6]. This makes infants with CKD particularly vulnerable for complications such as rickets, skeletal deformities, bone pain, and growth retardation. Ca deficiency may further worsen secondary hyperparathyroidism (SHPT), especially in conjunction with P deficiency, lead to mineralization abnormalities and rickets [7, 8]. These problems are compounded as the youngest of these infants depend largely on a milk-based diet, and commonly have feeding difficulties that may further complicate the management of CKD-MBD.

Pediatric guidelines have been published for bone and nutritional evaluation in children with CKD 2-5D [9, 10], but there are few studies on the diagnosis and management of CKD-MBD in neonates and infants, and therapeutic decisions are largely based on limited evidence. Here, we present clinical practice points addressing the diagnosis and management of CKD-MBD in children up to 2 years of age based on the available evidence, extrapolation from studies in older children where appropriate, and expert opinion of the European Society for Paediatric Nephrology (ESPN), CKD-MBD and Dialysis working groups (WGs) and the Pediatric Renal Nutrition Taskforce (PRNT).

## Methods

### The consensus development group

Two groups were assembled: a core working group and a Delphi panel. The core group comprised pediatric nephrologists and a pediatric kidney dietitian who were members of at least one of the above WG (i.e., ESPN dialysis, ESPN CKD-MBD, and/or PRNT), and who defined the scope of the project, formulated the PICO questions, performed a critical appraisal of the literature, rated the quality of evidence, GRADE-ed recommendations, conducted the Delphi process, and drafted the manuscript. The Delphi group included members of ESPN CKD-MBD and dialysis WGs, PRNT, and two neonatologists with a special interest in neonatal nephrology.

### Developing PICO questions

In order to give specific actionable advice, we developed PICO questions and formulated into actionable statements supported by the available evidence as follows:

- Patient (or population) includes children below 2 years with CKD or on dialysis, but three age ranges will be identified when necessary: neonates (0–1 month), 1–12 months, and 1–2 years. Indeed, while neonates correspond to a very specific group with distinct clinical and ethical issues [11–13], in infants less than 1 year kidney maturation, rapid growth and weaning may present additional issues that impact on MBD. We will also include infants born pre-term (with corrected gestational age) as there is an increased incidence of prematurity in infants with CKD [14, 15], and as nephrons continue to form up to 36 weeks of corrected gestational age [16–18].

CKD in infants is defined by persistent abnormal creatinine levels above the 97.5th percentile in the first 12 months of life [19, 20], and with estimated glomerular filtration rate (eGFR) < 90 mL/min per 1.73 m^2^ using the Schwartz formula thereafter [19, 21]. The assessment of kidney function in infants is highly challenging and beyond the scope of this guideline.

- Intervention may be “no action,” placebo, or an alternative therapeutic intervention.
- Comparison: Growth and biochemical markers were compared to healthy infants [22, 23], accounting for the effect of prematurity where relevant.
- Outcomes included growth, delayed motor milestones (e.g., walking and standing), bone disease (e.g., rickets, fractures, bowing), as well as the evolution of the biochemical parameters including Ca, P, bicarbonate (HCO_3_), parathyroid hormone (PTH), 25-hydroxyvitamin D (25(OH)D), and alkaline phosphatase (ALP) levels.

### Literature review and studies included

The PubMed database was searched until March 2022. We included the following articles: randomized controlled trials (RCTs), prospective studies (uncontrolled or observational) irrespective of number of patients, and retrospective studies including more than 5 cases, and registry data, all restricted to human studies in English, as summarized in Tables 1 and 2 [1–3, 5, 24–37].

**Table 1:** Review of the evidence for observational studies with an evaluation of MBD in infants under 2 years with CKD

Authors	Year	Ref	Population	Study design	Aim	Outcome
Rees et al.	2011	[24]	153 infants from 18 countries starting chronic peritoneal dialysis < 24 months	Registry study	Associations of feeding practices and other dialysis-related factors with length and weight gain in children	PTH levels 222 (85 to 529) pg/mL No data on associations of PTH with growth
Borzych et al.	2010	[26]	890 children from 24 countries	Registry study	Assessments of CKD-MBD in peritoneal dialysis	6% < 1 year and 16% < 1–5 years had hypophosphatemia 15% < 1 year and 21% 1–5 years had PTH 9 × above normal (KDIGO) Clinical and radiological symptoms markedly increased when PTH exceeded 300 pg/mL, the risk of hypercalcemia increased with levels below 100 pg/mL, and time-averaged PTH concentrations above 500 pg/mL were associated with impair longitudinal growth Associations were not analyzed separately in infants
Seikaly et al.	2006	[27]	5165 children with GFR < 75	Registry study	Correlates of short stature (< − 1.88) at entry to the registry	0–2 years had a mean height SDS of − 2.3 Ca, P, and PTH were NOT associated with short statures at entry
Schmitz et al.	2021	[74]	41 children, median age 1.1 year, range 0.5–8.0, CKD stage 3 to dialysis	Retrospective multi-center study	To evaluate enteral Ca intake and its association with PTH levels in pre-school children with CKD	Severe Ca deficiency found in 26% of children (with a significant variation between the first and second dietary data collection); 10% of children received enteral Ca above the upper target for age. Negative correlation between dietary calcium intake and PTH levels
Cansick et al.	2007	[28]	35 children aged 0.25 to 8.9 years	Retrospective single center study	Association of growth with PTH in children on dialysis	17 children under 2 years, the median change in HtSDS in the first year on dialysis was + 0.31 (range − 0.78 to 3.13) Significant association of change in HtSDS with ALP but no association of height with PTH in the study population Infants 0–2 years were not analyzed separately
Vidal et al.	2017	[1]	1063 infants 12 months or younger who initiated dialysis therapy in 1991 to 2013	Registry study	The impact of different dialysis modalities on clinical outcomes in young infants with chronic kidney failure	Mean PTH at dialysis initiation 496 (438 to 555) pg/mL similar in PD and HD
Shroff et al.	2006	[30]	98 children (61 boys), twenty-one < 1 year of age and 54 under < 5 years	Single center study	Outcome since 1984 of all children receiving chronic dialysis for > 3 months with a minimum follow-up of 5 (median 7.2) years	The measured intact PTH level at the start of dialysis was less than twice the ULN in 15 (16%) children as compared to a considerably improved control with 80 (88%) children having a PTH level within twice the upper limit of normal at final follow-up or pre-transplantation Separate analysis for infants not available
Shroff et al.	2003	[31]	18 children under 2 years of age received chronic hemodialysis (HD)	Single center study	Morbidity and outcome of chronic HD in children under 2 years of age	Intact PTH was less than twice the upper limit of the reference range for the assay in 41% of the children when HD was started and in 69% (*n* = 11) after 3 months
Paglialonga et al.	2016	[32]	21 children < 2 yrs at initiation of HD	Registry study	Outcomes in HD	PTH 169 (76–628) pg/mL at baseline, 336 (136–1088) at 6 months, and 207 (64–900) at 12 months
Ledermann et al.	2000	[33]	20 infants with a mean age of 0.34 year (range, 0.02–1 year)	Single center study	Outcome in PD	Intact PTH was less than twice the upper limit of the reference range in 58% of the infants (*n* = 12) after 6 months of PD and 100% (*n* = 14) after 1 year, with the values falling within the normal range in 79% Associations with growth were not assessed
Dachy et al.	2020	[5]	17 infants with age 2.6 (0.1;5.9) months at peritoneal dialysis initiation	Retrospective study	Experience of two French tertiary pediatric nephrology centers on 17 infants who began chronic PD before 6 months of age	Before PD, median PTH levels were 212 (6;799) ng/L, decreasing to 130 (23;732) ng/L 3 months after the beginning of PD, and reaching 123 (44;1540) ng/L at the end of PD (*p* = NS between PD initiation and the end of PD) Associations of PTH with growth were not assessed
Vidal et al.	2012	[34]	84 infants who started CPD at < 1 year of age	Registry study	Growth data analysis was performed only in infants with complete auxological parameters at 0, 6, and 12 months of follow-up	No significant association of catch-up growth with PTH intact PTH at initiation of dialysis was 508 (10–1760) ng/L at t0 and 351 (33–1650) at t12 in those with catch-up growth vs. 683 (163–2000) at t0 and 398 (81–3500) at t12 in those without catch-up growth

**Table 2:** Review of the evidence for interventional studies with an evaluation of MBD in infants under 2 years with CKD

Authors	Year	Ref	Population	Study design	Aim	Outcome
Arenas Morales et al.	2019	[35]	10 young pediatric patients median age 18 months (IQR 6–36), CKD5 and dialysis	Case series	Severe sHPT with intact parathyroid hormone (iPTH) levels ≥ 500 pg/mL refractory to standard therapy for > 30 days receiving cinacalcet	Linear growth improved significantly during cinacalcet therapy in 80% (ΔSDS − 0.62 ± 1.2 versus + 0.91 ± 1.4; *p* < 0.005). By multiple regression analysis, the primary determinants of growth were concurrent treatment with growth hormone and age < 2 years (*R*^2^ = 89.6%; *p* < 0.001) Average starting dose of 0.7 ± 0.2 mg/kg/day; median effective dose required to reach PTH goal: 2.8 mg/kg/day (IQR 2.0–3.1); 4 patients with asymptomatic hypocalcemia
Joseph et al.	2019	[36]	18 patients (9 male) less than 5 years, mean age at initiation of cinacalcet was 2.3 years (range 8 months–4.5 years); 13 PD and 5 HD	Single center case series	Efficacy and safety of cinacalcet for treatment of secondary hyperparathyroidism in chronic dialysis patients younger than 5 years	Significant improvement in linear growth was observed while on cinacalcet and growth hormone; average starting dose of cinacalcet 0.55 mg/kg/day
Saarinen et al.	2007	[37]	16 bilaterally nephrectomized infants with congenital nephrosis of the Finnish type (median age 0.54 years) on peritoneal dialysis	Single center case series	9 were receiving intermittent 1-alpha calcidol therapy and 7 daily with target serum parathyroid hormone (PTH) level 2–3 times the upper limit of normal (ULN)	Intermittent or daily vitamin D analog therapy prevents secondary hyperparathyroidism equally well, but intermittent therapy might be more favorable for growth Only a weak but significant correlation between alkaline phosphatase and the change in HtSDS from 3 to 6 months
Schmitt et al.	2003	[64]	24 prepubertal children with CKD; among them 2 infants were included	Randomized multi-center study	To compare the effect of daily versus twice-weekly calcitriol on PTH and growth	No difference in PTH, calcium, and phosphate levels in the two arms. Identical growth over 1 year

### Grading system

We applied the grading system from the AAP [38], as previously described [39], and illustrated in Supplemental Fig. 1. Delphi participants were required to complete an e-questionnaire, stating their level of agreement for all 34 clinical practice points on a 5-point scale (strongly disagree, disagree, neither agree/disagree, agree, strongly agree). A consensus level of at least 70% was required, and statements that achieved below 70% required rewording. We obtained 20 responses to the validation questionnaire.

## Clinical practice points

### How to assess CKD-MBD in infants?

#### Clinical evaluation

1.1.1 We suggest physical examination for clinical signs of CKD-MBD in infants with the frequency of assessment based on the infant’s corrected gestational age, stage of CKD, comorbidities, and severity of MBD (level X, moderate recommendation).
1.1.2 We suggest regular monitoring of growth (weight, length, and head circumference), and plotted serially on centile growth charts (level C, weak recommendation).
1.1.3 In children with inherited disorders that affect bone health, we suggest specific follow-up tailored to their underlying kidney disease (level C, weak recommendation).

##### Rationale

A complete history divided into prenatal-antenatal and postnatal clinical history should be taken once at the first visit, while the history of CKD-MBD symptoms (e.g., gait, limping, and pains) should be taken at each follow-up visit. Growth, including weight, length, and head circumference, should be carefully followed, notably by plotting serial measurements on age- and sex-appropriate growth charts [10].

Comorbidities that can affect motor milestones should be kept in mind. Caregivers must be asked about the achievement of age-appropriate developmental milestones including standing and walking ages, and any difficulties in routine daily physical activity. A detailed musculo-skeletal examination focusing on range of motion in joints, the presence of any point tenderness or pain, limping, and bony deformities is recommended, as summarized in Supplemental Table 1 [9]. Infants should be evaluated for clinical signs of rickets, i.e., widening of the forearm at the wrist due to widened metaphyses, thickening of the costochondral junctions, Harrison’s groove, and long bone bowing once they start weight bearing.

Infants with inherited kidney disorders that affect bone health, and notably phosphate-wasting tubulopathies (including but not restricted to nephropathic cystinosis) and primary hyperoxaluria (PH), should be followed and managed accordingly [40–42].

#### Biochemical evaluation

1.2.1 We suggest measuring biomarkers of CKD-MBD (Ca, P, ALP, PTH, 25(OH)D, HCO_3_) with frequency of assessment based on the infant’s (corrected) age, stage of CKD, underlying disease, and the presence and severity of MBD (level C, moderate recommendation).
1.2.2 We suggest to measure ionized Ca levels where available (level C, weak recommendation).

##### Rationale

Serum bone biomarkers including Ca (ionized Ca, where available), P, ALP, PTH, and 25(OH)D should be monitored regularly depending on CKD stage, underlying kidney disease, and presence and severity of MBD-related clinical and biochemical abnormalities, as suggested in Table 3. The first year of life and puberty are periods of rapid growth, requiring the highest Ca intake [43]. These higher Ca requirements are reflected in higher normal values for serum total Ca, particularly during infancy, and reach adult levels by 5 years of age. Age-dependent normal values as shown in Table 4 [9, 44, 45] should be used.

**Table 3:** Proposed minimal frequency of clinical, biochemical, and radiological assessment (in months) of CKD-MBD in infants by CKD stage and age

		Mild CKD	Moderate CKD	Severe CKD and dialysis
Age 0–1 years	Anthropometry and clinical evaluation	1–3	0.5–2	0.25–1
	Biochemicals (Ca, P, bicarbonate, PTH, ALP)	3–6	1–3	0.25–1
	Biochemicals (25OH)	6	3–6	3
	X-rays^a^	Only in case of clinical signs	Only in case of clinical signs	Only in case of clinical signs
Age 1–2 years	Anthropometry and clinical evaluation	3–6	1–3	0.5–2
	Biochemicals (Ca, P, bicarbonate, PTH)	3–6	1–3	0.5–1
	Biochemicals (25OH)	6–12	3–6	3
	X-rays^a^	Only in case of clinical signs	Only in case of clinical signs	Only in case of clinical signs

The clinical and nutritional assessment may even be more frequent if needed, especially in the youngest infants and in those with kidney failure. ^a^We suggest plain X-rays of areas of rapid growth such as wrist, knee, and ankle in infants with clinical or biochemical features of rickets, gait abnormalities, limping, bone pain, and suspected atraumatic fractures

**Table 4:** Age-specific reference ranges of the main parameters of phosphate/calcium metabolism in term infants, adapted from [9, 44, 45]

	iCa mmol/L	Ca mg/dL	Ca mmol/L	P mg/dL	P mmol/L	ALP IU/L	Albumin g/L	Creatinine mg/dL	Creatinine µmol/L	PTH	25 OH-D nmol/L	25OH-D ng/mL
0–14 days	1.22–1.40	8.5–11.0	2.13–2.75	5.6–10.5	1.81–3.39	90–273	33–45	0.32–0.92	28–81	No assessment before 4 weeks of life	75–120	30–48
15–30 days	1.22–1.40	8.5–11.0	2.13–2.75	4.8–8.4	1.55–2.71	134–518	28–47	0.10–0.36	9–32	No assessment before 4 weeks of life	75–120	30–48
30 days to 6 months	1.22–1.40	8.5–11.0	2.13–2.75	4.8–8.4	1.55–2.71	134–518	28–47	0.10–0.36	9–32	Mild CKD: normal levels Moderate CKD: 1–2 ULN Advanced CKD/dialysis: 2–3 ULN	75–120	30–48
6 months to 1 year	1.20–1.40	8.5–11.0	2.13–2.75	4.8–8.4	1.55–2.71	134–518	28–47	0.10–0.36	9–32	Mild CKD: normal levels Moderate CKD: 1–2 ULN Advanced CKD/dialysis: 2–3 ULN	75–120	30–48
1 to 2 years	1.22–1.32	9.2–10.5	2.30–2.63	4.3–6.8	1.39–2.20	156–369	38–47	0.10–0.36	9–32	Mild CKD: normal levels Moderate CKD: 1–2 ULN Advanced CKD/dialysis: 2–3 ULN	75–120	30–48

Results are presented as lower and upper limit. For calcium, the conversion factor from mg/dL to mmol/L is to divide by 4.0. The calculation formula for corrected calcium (CaC, mmol/L) using measured calcium (mmol/L) and serum albumin (g/L) is the following: CaC = Ca − 0.25 × (serum albumin − 40). If serum albumin is not available, CaC may be calculated with protidemia (g/L) with the following formula: CaC = Ca/(0.55 + P/16). For phosphate, the conversion factor from mg/dL to mmol/L is to divide by 3.1. For creatinine, the conversion factor from mg/dL to µmol/L is to multiply by 88.4. In the CALIPER database, albumin was assessed with bromocresol green and creatinine with an enzymatic assay. *ULN*, upper limit normal for the local assay

Mineralization defects were noted in 29% of children in CKD stage 2, and in nearly 80% in those with CKD stages 4–5, in a cohort of 52 pre-dialysis children between 2 and 21 years of age, at a mean age of 12.2 ± 5.2 years [46]. In children on peritoneal dialysis (PD), deficient bone mineralization was more common than turnover abnormalities in a cohort of 161 children between 0.7 and 20 years of age (among them 21 were under 5 years) [8]. Similarly, a high P demand should be considered in infants attributable both to rapid skeletal growth and high renal tubular and dialysate phosphate losses. Chronic hypocalcemia and/or hypophosphatemia can develop in infants receiving intensified dialysis regimens such as frequent daily or nocturnal hemodialysis (HD), leading to impaired bone mineralization and rickets [47]. In addition, patients with phosphate-wasting tubulopathies (including nephropathic cystinosis) may have low fibroblast growth factor 23 (FGF23) and PTH levels despite advanced CKD, and significant metabolic bone disease characterized by hypophosphatemic rickets in infancy, bone pain, and deformities [40, 48].

Because of the specific kinetics of PTH and vitamin D metabolism during the neonatal period, the interpretation of these two biomarkers, and especially PTH, is challenging during the first weeks of life. Indeed, after birth, when mineral transfer from the placenta is interrupted and PTH-related peptide is not secreted any more, there is a transition from fetal to neonatal regulation characterized by a fall of serum Ca during the first 24–48 h of life and a rise of P, leading to increased PTH and 1–25(OH)_2_ vitamin D levels [49]. The fetal parathyroid gland starts secreting PTH at 10 weeks of gestation, and maternal PTH does not cross the placental barrier [49]. In contrast, calcitriol levels in the neonate reflect maternal levels, and there are early changes because of the interruption of its secretion by the placenta [49]. Serum mineral levels normalize over several days to almost-adult values, regulated by PTH, calcitriol, calcitonin and FGF23, and also dependent on enteral sources of minerals [49]; several factors also affect gastro-intestinal absorption of minerals in neonates such as type of milk, enteral phosphorus, and vitamin D supplementation. As a consequence and as illustrated in Table 4, PTH levels should not be assessed before 4 weeks of life and should be interpreted cautiously; the timing of first PTH measurement will depend on the child’s overall health context, and should not happen before 1 month in case of advanced CKD but not later than 3 months in case of mild CKD. Preterm infants are prone to vitamin D deficiency due to incomplete transplacental transfer during the third trimester, low body stores, low vitamin D in parenteral nutrition, decreased absorption, and negligible sun exposure during hospital stay [50], in addition to the classical risk factors of vitamin D deficiency in newborns such as use of breast milk, ethnicity, and/or vitamin D deficiency in the mother. Thus, an earlier assessment of 25(OH)D levels should be performed in the first 2 weeks of life, because of the high proportion of native vitamin D deficiency at birth, both in term and especially in preterm infants [50], but also because with standard vitamin D supplementation preterm infants may display 25(OH)D under- or over-dosing at term-corrected age [51]. Some studies in preterm infants without CKD have shown the importance of an early monitoring of 25(OH)D levels so as to adapt supplementation [52]. After the first evaluation, 25(OH)D levels should not be measured more than quarterly [45], except for preterm infants receiving more than 1000 IU per day. More generally, preterm infants are prone to mineral bone disease, and several risk factors have been identified such as medications (steroids, diuretics, caffeine, antacids) and morbidities (broncho-pulmonary dysplasia, necrotizing enterocolitis, and cholestasis) [53, 54], that may also be present in CKD preterm infants.

Bicarbonate levels may be lower in infants: bicarbonate threshold is 18 mmol/L in preterm infants (being as low as 14 mmol/L in the extremely low gestational age neonate) and 21 mmol/L in term infants, reaching adult levels (i.e., 24–26 mmol/L) only after the first post-natal year [55], and more likely around 2 years of age [56]. Even though evidence is scarce in infants, there is experimental evidence showing that acidosis is deleterious for bone by inhibiting osteoblastic mineralization and stimulating both osteoclastic differentiation and resorption [57, 58]. In older children, acidosis (bicarbonate levels < 22 mmol/L) is shown to be a risk factor for CKD progression [59], and correcting acidosis prevents the progression of CKD [60]. As such, we may hypothesize that compensation of metabolic acidosis for bone and CKD progression is at least as important in vulnerable infants with rapid development as in older children. Also, metabolic alkalosis may develop in infants who have frequent vomiting; this might affect the interpretation of serum calcium levels. Hence, we recommend that ionized calcium levels are measured when possible so that hypocalcemia can be detected and managed appropriately. It remains to be determined whether blood pH may be useful for CKD-MBD evaluation and management in infants.

#### Radiological evaluation

1.3.1 Do not perform routine X-rays in infants with CKD (level D, weak recommendation).
1.3.2 We suggest plain X-rays in infants with clinical suspicion of rickets or other bone involvement (level D, weak recommendation).
1.3.3 We suggest an individualized approach to radiological monitoring in infants with genetic diseases with specific bone involvement (level C, moderate recommendation).

##### Rationale

As previously detailed in the guidelines of bone evaluation in pediatric CKD, X-rays should not be performed on a routine basis [9]. There may be a lower threshold for performing X-rays in infants than in older children, but evidence is very scarce. Plain X-rays can visualize common radiographical signs of CKD-MBD including osteopenia, erosions and radiolucent zones in metaphyses, and signs of rickets such as an increased thickness of the growth plates of long bones, with irregular, hazy appearance at the diaphyseal line, and rachitic rosaries [9]. Thus, in case of suspicion of inadequate control of CKD-MBD and early signs in infants, we suggest performing X-rays, preferentially of areas of rapid growth such as wrist, knee, and ankle in the absence of overt clinical symptoms. The use of “low-dose” radiographs and scans whenever possible should be favored. Other imaging techniques, including dual-energy X-ray absorptiometry (DXA), magnetic resonance imaging (MRI), or ultrasounds, are not recommended for routine monitoring, but may be used for research purposes or special circumstances such as the diagnosis of osteomyelitis. Some authors have reported an interest in quantitative ultrasounds in this age group to assess metabolic bone disease in preterm infants [61], but this was not evaluated in CKD patients.

Last, the hand/wrist method of Greulich and Pyle for bone age evaluation is suboptimal in infants because the ossification centers of the hand and wrist do not change significantly during infancy; as such, in children under the age of 3 the bone maturation process is better characterized by the X-rays of the knee [62].

### What targets optimize management of CKD-MBD in children under 2 years of age?

#### Biochemical targets

2.1 We suggest maintaining serum Ca, P, ALP, and HCO_3_ within the age-related normal ranges (level D, weak recommendation).
2.2 We suggest maintaining PTH levels within the CKD-stage dependent target ranges (level D, weak recommendation).
2.3 2.3 We suggest maintaining 25(OH)D within the target range defined in older children (level C, weak recommendation).
2.4 We recommend that therapeutic decisions be based on trends rather than on a single laboratory value, considering all available CKD-MBD assessments (level X, strong recommendation).

##### Rationale

Several registry and single center studies reported on PTH levels in infants on dialysis. Data from the International Pediatric Dialysis Network (IPPN) registry on 890 children on peritoneal dialysis from 24 countries have rather found a negative effect on longitudinal growth of high PTH levels above 500 pg/mL [26]. Less than 20% of children < 5 years had PTH within the KDOQI limits in the IPPN registry [26], and PTH above twice the upper normal limit was found in 39% of infants < 1 year and 54% of children between 1 and 5 years [26]. Similarly, 59% of children under 2 years displayed PTH above twice the upper normal limit in a single center study including 20 infants on dialysis < 1 year [33]. Most studies showed regression of PTH values after initiation of dialysis in infants [1, 2, 5, 24, 28, 31, 32, 34]. However, these studies have not assessed the associations of temporal trends in PTH levels and combination of biomarkers with growth and/or bone disease, which are considered to better reflect underlying osteodystrophy based on evidence in older CKD children and adolescents [9]. Some studies have suggested a positive relation between PTH and growth, with likely deleterious effects of active vitamin D therapy on growth [63, 64].

In infants starting dialysis before the age of 12 months, there was no significant difference in PTH levels between hemodialysis (HD) and PD [1]. In the Italian registry which included 84 infants, PTH levels at dialysis initiation and 12 months later did not differ between infants who displayed catch-up growth as opposed to those who failed to present catch-up growth [34]. Last, hemoglobin levels have been associated with PTH levels, reflecting possible bone marrow suppression because of the fibrosis observed in SHPT [65]. However, there is still no clear consensus on the target PTH level [66–68]. PTH alone does not accurately reflect underlying renal bone disease; high bone turnover may occur at lower thresholds than currently recommended PTH targets [9]. The ideal target according to CKD stage would be a balanced approach avoiding high, but also too low, PTH levels that could be associated with adynamic bone disease and vascular calcifications. This is especially true for infants in whom long-term consequences may develop and progress from an early age [69, 70].

The only study including infants (among older children) with CKD before dialysis failed to show associations of Ca, P, and PTH with clinical symptoms of CKD-MBD and short stature at entry [27]; interestingly, infants of 0–2 years had the lowest height standard deviation score (SDS) at study entry, but the analysis for the aforementioned associations was not separated by age groups [27]. Only half of the infants < 1 year and children 1–5 years had Ca and P levels within the KDOQI recommendations in the IPPN registry [26]. The evidence of targeting serum Ca and P to normal values for age derives mainly from studies in older CKD patients as well as non-CKD infants and preterm infants [9]. It has been proposed that an individualized approach using biological age (height age) would be more appropriate in this age group [71]. In addition to CKD-MBD, metabolic bone disease of prematurity may further complicate the management of preterm infants with CKD [72].

### The dietary management of CKD-MBD in infants

#### Consensus statements:

3.1 We suggest that the dietary Ca and P intake in infants with CKD should be assessed regularly (level C, weak recommendation).
3.2 We suggest that the total Ca intake from feed, food, and medications is within the suggested dietary intake (SDI) in infants with CKD. The Ca requirement may need to exceed twice the SDI in infants with rapid growth or those with mineral depleted bone, with careful monitoring (level C, weak recommendation).
3.3 We suggest that the dietary P intake from feed and food is within the SDI for age (level C, weak recommendation).
3.4 A phosphate restricted diet must not compromise protein or calcium intake, with addition of phosphate binders as required (level X, strong recommendation).
3.5 We recommend using preterm infant nutritional requirements as a guide for preterm infants with CKD, adjusting their intake according to growth and biochemistry (level D, weak recommendation).

#### Rationale

A dietary assessment of infants with CKD should include energy, protein, and micronutrients aiming for the SDIs for calcium and phosphate, as outlined in previous recommendations [7, 10, 73–75] from the PRNT and illustrated in Table 5 [7]. A specialist pediatric kidney dietitian or healthcare professional who has the necessary skills and training should carry out the dietary assessment [76]. The optimal frequency of dietary assessment depends on the infant’s CKD stage, growth, biochemical control, and any feeding-related problems. We suggest a dietetic contact at least every 4 weeks and taking a diet history at least every 8 weeks [10, 56].

**Table 5:** Suggested dietary intakes (SDIs) per day for Ca and P in infants with CKD2-5D, adapted from [7]

Age	SDI calcium (mg/d)	SDI phosphate (mg/d)
0– < 4 months	220	120
4– < 12 months	330–540	275–420
12–24 months	450–700	250–500

Recommendations on dietary management of Ca and P in children with CKD stages 2–5 and on dialysis have been published [7], as well as on nutritional management of infants [75]. An upper limit for Ca intake for healthy children of different ages has not been defined; however, 200% of the SDIs has been suggested as a safe upper limit for Ca intake from diet and phosphate binders in CKD [7, 47]. Of note, the Ca intake from the diet may not provide sufficient Ca [74], particularly as most Ca-rich foods also contain large amounts of P, and are likely to be restricted in CKD patients [77]. In some situations, such as during periods of rapid catch-up growth, when there is a late presentation with untreated CKD, or in infants on daily hemodialysis, greater Ca intake than the SDI may be required [24, 78]. Also, intestinal Ca absorption may be impaired in vitamin D-deficient infants [45]. When there is either persistent hyperphosphatemia or persistent SHPT in the presence of normal Ca levels, limiting dietary P intake to the lower limit of the SDI may be required [76]. Care needs to be taken not to compromise the intake of protein and other nutrients when restricting dietary P, with the use of P binders to control P and PTH levels if necessary. Ca supplements may be necessary to achieve an adequate intake of Ca. Infants with tubular disorders and those on intensified dialysis regimens are likely to have phosphate wasting and will need phosphate supplementation. All standard and most specialized infant formulas have a higher concentration of Ca relative to human milk (because of a lower bioavailability of Ca from formulas) [79], but a lower concentration than cow’s milk (Supplemental Table 2) [75].

Breastfeeding is the preferred method for feeding an infant with CKD [75]. If an infant is drinking infant formula, vitamin D intake from infant formula should be assessed (Supplemental Table 2), and any vitamin D supplementation adjusted accordingly [45].

Given the high Ca and P requirements of preterm infants, the European Society for Paediatric Gastroenterology, Hepatology and Nutrition (ESPGHAN) recommends 120–140 mg/kg/day of Ca, 60–90 mg/kg/day of P, Ca-P ratio of 1.5–2.0:1, and 800–1000 IU/day of vitamin D [80]. The recommendations relate to ranges of enteral intakes for stable-growing preterm infants up to a weight of 1.8 kg. No recommendations are made for infants < 1 kg as data are lacking. ESPGHAN/ESPN/ESPR/CSPEN guidelines are available for preterm infants requiring parental nutrition (PN) [81, 82]. Chinoy et al. recommend that all infants with a birth weight less than 1500 g should be screened for metabolic bone disease of prematurity and the treatment path based on plasma PTH levels [53]. These enteral and PN recommendations may be considered for preterm infants with CKD as a guide but each infant needs to be assessed individually, adjusting their nutrition and supplementation according to growth and biochemical parameters. In infants who are unable to meet their nutritional requirements orally, supplemental or exclusive enteral tube feeding should be commenced [73].

Complementary foods should be introduced in an age-appropriate form around 6 months of age for term infants (but not before 4 months corrected age) [83]. All infants should be assessed individually for their readiness for solids. Advice on the introduction of complementary foods must include controlling the intake of higher P foods such as dairy and animal protein. In contrast, a plant-based diet that is rich in phytates will reduce phosphate absorption. Processed foods that are likely to contain inorganic highly bioavailable P additives should be avoided as much as possible [7].

### Specific aspects of the medical management of MBD in infancy

#### Native vitamin D

4.1.1 We suggest that all newborns, including preterm infants, with CKD receive vitamin D supplements from birth (level C, moderate recommendation).
4.1.2 We suggest that serum 25(OH)D concentrations are kept within a target range of 75–120 nmol/L (level D, weak recommendation).

#### Calcium supplementation and calcium in the dialysate

4.2.1 We recommend Ca supplementation in case of hypocalcemia (level X, strong recommendation).
4.2.2 We suggest oral Ca supplementation in case of persistently high PTH levels, provided P levels are controlled (level D, weak recommendation).
4.2.3 Use intravenous Ca to correct serum Ca levels in acute hypocalcemic emergencies (level X, strong recommendation).
4.2.4 We suggest increasing dialysate Ca concentrations in order to keep serum Ca (ionized Ca where available) levels in the normal range in infants undergoing dialysis (level D, weak recommendation).
4.2.5 We recommend avoiding vitamin A supplementation in infants with CKD (level X, strong recommendation).

#### Phosphate binders and phosphate supplementation

4.3.1 Maintain P levels within the normal range for age, by adapting nutrition first but without compromising protein intake (level C, moderate recommendation).
4.3.2 Introduce P binders if serum P is not controlled with optimized nutritional management (level C, moderate recommendation).
4.3.3 We suggest using Ca-based P binders as first-line therapy (level D, weak recommendation).
4.3.4 We suggest considering sevelamer carbonate in infants with hypercalcemia (level D, weak recommendation).
4.3.5 We suggest providing P supplementation after optimization of nutritional phosphate intake in case of persistent hypophosphatemia (level D, weak recommendation).

#### Management of secondary hyperparathyroidism

4.4.1 We suggest starting active vitamin D in the lowest dose to achieve target PTH and normal calcium levels (level C, moderate recommendation).
4.4.2 We recommend giving native vitamin D and active vitamin D analogs directly by mouth, and avoiding nasogastric or gastrostomy tubes (level X, strong recommendation).
4.4.3 We recommend considering initiation or optimization of dialysis in infants with persistently uncontrolled SHPT and/or hyperphosphatemia despite optimized nutritional and medical management (level C, moderate recommendation).
4.4.4 Cinacalcet may be considered with extreme caution in infants on dialysis who have persistent and severe hyperparathyroidism in the presence of high or high-normal calcium levels, despite optimized conventional management, including active vitamin D (level D, weak recommendation).
4.4.5 Parathyroidectomy may be considered as a last resort treatment when all medical management fails to control SHPT (level D, weak recommendation).

##### Rationale-Native vitamin D

The recommended daily vitamin D intake for healthy infants without risk factors for vitamin D deficiency amounts to 400 IU during the first year of life, as stated in the 2013 ESPGHAN guidelines [84]. Even though a daily supply of 400 IU vitamin D may not be sufficient in children older than 12 months, ESPGHAN did not propose a daily dose after 1 year, rather encouraging countries to adopt policies to improve vitamin D status through food fortification, dietary recommendations, and judicious sun exposure, with oral supplementation of vitamin D to be considered beyond 1 year of age depending on local circumstances [84]. In accordance with the European Food Safety Authority, the upper limit of safety is set at 1000 IU/day for infants and 2000 IU/day for children aged 1 to 10 years [85]. Higher doses may be required in infants with CKD, as previously demonstrated in older children with CKD [86, 87].

A few RCTs on native vitamin D supplementation have been performed in preterm infants [88–95], showing that a daily dose between 600 and 1000 IU per day is sufficient to prevent rickets. We therefore recommend this procedure also for infants with CKD for prophylaxis of rickets. The “intensive replacement phase” may follow the 2017 European guidelines, providing infants less than 1 year with 600 IU per day and infants older than 1 year a regimen depending on their baseline 25(OH)D levels [45]. However, the evidence for these doses and weight-related vitamin D dosages is low, since the RCTs on treatment with native vitamin D in children did not include children below 2 years of age [86, 87, 96]. In children with CKD stages 2–5 (no infants) started on supplementation with native vitamin D, body surface area (BSA)-related vitamin D dosage and PTH and phosphate levels at baseline were independently associated with final serum 25(OH)D levels by multiple linear regression analysis. Likewise, the change in 25(OH)D levels was positively associated with vitamin D dosage [97]. Model-based simulations showed that current dosing recommendations for cholecalciferol can be optimized using a weight-based dosing strategy [98].

### Calcium supplementation and calcium in the dialysis fluid

Infants on dialysis are much more likely to need a higher dialysate Ca concentration than older children, especially if enteral intake (from diet and medications) cannot maintain normal serum Ca levels to control PTH. One additional point that one should not forget is the risk of hypervitaminosis A with some formulas (especially in case of parenteral nutrition) and subsequent hypercalcemia; some authors even discuss monitoring vitamin A levels as part of routine nutritional assessments in pediatric CKD [99, 100].

### Phosphate binders and phosphate supplementation

Physiological serum P concentrations are substantially higher in infants compared to older children [44]. Higher serum P concentrations may be acceptable in infants with CKD-MBD, even though the impact is uncertain. Two RCTs have been performed in CKD children to compare sevelamer hydrochloride with calcium carbonate [101, 102], but only one included infants under 2 years [102]. P control was similar with the two compounds; however, an increased risk of hypercalcemia was found with calcium acetate, as opposed to an increased risk of acidosis with sevelamer hydrochloride [102]. The latter side effect has not been observed with sevelamer carbonate [103]. In the youngest infants, there are practical limitations, notably the multiple feeding episodes during 24 h and the risk of blocking nasogastric or gastrostomy tubes with the compounds. It was therefore proposed to add binders directly into the formula and then feed the treated supernatant [104, 105], but this procedure may modify the composition of the formula and decrease the content of micronutrients [106]. This technique should not be used in countries where adapted renal formulas are available or where experienced kidney dietitians can give advice. Of note, breastfeeding is low in phosphate.

### Active vitamin D

We refer the reader to the previously published guidelines on active vitamin D in CKD children [107]. Both daily and intermittent (thrice weekly) alfacalcidol prevented the development of SHPT in 18 infants with congenital nephrotic syndrome undergoing PD (median age 0.54 years) [37]. Importantly, both native and active vitamin D should be given directly by mouth, since they have the potential to stick to plastic tubes. Caregivers should be regularly reminded of this, especially in case of uncontrolled SHPT.

### Calcimimetics

Calcimimetics are not licensed in children under 3 years of age [39], but some authors have reported their use in infants with advanced CKD or on dialysis, however with greater average doses at initiation than the current guidelines in older children (0.2 mg/kg/day to begin, maximum 2.5 mg/kg/day), as illustrated in Table 2 [35, 36]. Cinacalcet in combination with recombinant human growth hormone (rhGH) treatment was associated with a significant improvement of standardized height in children undergoing dialysis and aged less than 5 years; treatment was effective in decreasing PTH levels and well tolerated [36]. In the second study, cinacalcet was given to 10 infants (median age 18 months) on dialysis, with a significant median overall decline in PTH of 82% from baseline by 6 months; the median effective dose required to reach such a control was 2.8 mg/kg/day (IQR 2.0; 3.1) [35]. Linear growth significantly improved during cinacalcet therapy [35]. Thus, calcimimetics may be considered as an alternative to parathyroidectomy in individual cases after informed consent of the family, provided a close follow-up of ionized Ca and Ca levels and the subsequent risk of hypocalcemia. A recent European survey was performed to evaluate the use of cinacalcet in dialysis children below 3 years of age. In this study, 22 patients received cinacalcet at a median age of 28 (15–28) months and a median starting dose of 0.4 (0.2–0.8) mg/kg/day. Cinacalcet was described as giving an efficient control of PTH levels. Despite the fact that overall median calcium levels remained stable, three infants nevertheless developed hypocalcemic episodes < 2.0 mmol/L, of which one child suffered from a hypocalcemia-related convulsion, due to non-adherence to calcium substitution [108]. Interestingly, precocious puberty was reported in two children, as already described in older children [109].

### Parathyroidectomy

One paper reported the outcomes of parathyroidectomy in 18 CKD children, among them two patients who were below 2 years of age; results showed that it is an effective procedure with long-term control of serum PTH, Ca, and P [110, 111]. Postoperative complications comprised one episode of hypocalcemic seizure due to non-adherence with oral Ca supplements, one hemopericardium in a patient with thoracic adenoma, and transient bilateral vocal cord palsy, thus highlighting the non-negligible risks of such a procedure. The two infants experienced no complications. Thus, parathyroidectomy may be considered in case of SHPT, but only as a last resort, when all other medical management including calcimimetics have failed. In infants, the risk of severe hypocalcemia and growth retardation following parathyroidectomy are very high and sub-total parathyroidectomy may be considered when all medical management fails.

## Conclusions

Due to rapid bone growth and difficulties with nutrition, an adequate control of CKD-MBD in infants is particularly challenging. Here, we present 34 CPPs on diagnosis and management of CKD-MBD in infants under 2 years of age, as summarized in Table 6. We also include suggestions for daily practice. The practice points developed in previous guidelines of the working groups that are also valid for this age group were not considered separately [7, 9, 10, 39, 45, 73, 107, 112]. Given that there are very few high-quality studies to guide evidence-based practice, these statements must be carefully considered by the treating physician and adapted to individual patient needs as appropriate. We also propose future research topics in the field (Table 7).

**Table 6:** Summary of the 34 consensus statements with their respective grading

PICO question	Consensus statement
1 How to assess CKD-MBD in infants under 2 years of age?	1.1 Clinical evaluation 1.1.1 We suggest physical examination for clinical signs of CKD-MBD in infants with the frequency of assessment based on the infant’s corrected gestational age, stage of CKD, comorbidities, and severity of MBD (level X, moderate recommendation) 1.1.2 We suggest regular monitoring of growth (weight, length, and head circumference), and plotted serially on centile growth charts (level C, weak recommendation) 1.1.3 In children with inherited disorders that affect bone health, we suggest specific follow-up tailored to their underlying kidney disease (level C, weak recommendation) 1.2 Biochemical evaluation 1.2.1 We suggest measuring biomarkers of CKD-MBD (Ca, P, ALP, PTH, 25(OH)D, HCO_3_) with frequency of assessment based on the infant’s (corrected) age, stage of CKD, underlying disease, and the presence and severity of MBD (level C, moderate recommendation) 1.2.2 We suggest to measure ionized Ca levels where available (level C, weak recommendation) 1.3 Radiological evaluation 1.3.1 Do not perform routine X-rays in infants with CKD (level D, weak recommendation) 1.3.2 We suggest plain X-rays in infants with clinical suspicion of rickets or other bone involvement (level D, weak recommendation) 1.3.3 We suggest an individualized approach to radiological monitoring in infants with genetic diseases with specific bone involvement (level C, moderate recommendation)
2 What are the biochemical targets so as to optimize management of CKD-MBD?	2.1 We suggest maintaining serum Ca, P, ALP, and HCO_3_ within the age-related normal ranges (level D, weak recommendation) 2.2 We suggest maintaining PTH levels within the CKD-stage dependent target ranges (level D, weak recommendation) 2.3 We suggest maintaining 25(OH)D within the target range defined in older children (level C, weak recommendation) 2.4 We recommend that therapeutic decisions are based on trends rather than on a single laboratory value, taking into account all available CKD-MBD assessments (level X, strong recommendation)
3 What are the targets of dietary control of CKD-MBD in this age group?	3.1 We suggest that the dietary Ca and P intake in infants with CKD should be assessed regularly (level C, weak recommendation) 3.2 We suggest that the total Ca intake from feed, food, and medications is within the suggested dietary intake (SDI) in infants with CKD. The Ca requirement may need to exceed twice the SDI in infants with rapid growth or those with mineral depleted bone, with careful monitoring (level C, weak recommendation) 3.3 We suggest that the dietary P intake from feed and food is within the SDI for age (level C, weak recommendation) 3.4 A phosphate restricted diet must not compromise protein or calcium intake, with addition of phosphate binders as required (level X, strong recommendation) 3.5 We recommend using preterm infant nutritional requirements as a guide for preterm infants with CKD, adjusting their intake according to growth and biochemistry (level D, weak recommendation)
4 What are the specificities of therapeutic management in this age group?	4.1 Native vitamin D 4.1.1 We suggest that all newborns, including preterm infants, with CKD receive vitamin D supplements from birth (level C, moderate recommendation) 4.1.2 We suggest that serum 25(OH)D concentrations are kept within a target range of 75–120 nmol/L (level D, weak recommendation) 4.2 Calcium supplementation and calcium in the dialysate 4.2.1 We recommend Ca supplementation in case of hypocalcemia (level X, strong recommendation) 4.2.2 We suggest oral Ca supplementation in case of persistently high PTH levels, provided P levels are controlled (level D, weak recommendation) 4.2.3 Use intravenous Ca to correct serum Ca levels in acute hypocalcemic emergencies (level X, strong recommendation) 4.2.4 We suggest increasing dialysate Ca concentrations in order to keep serum Ca (ionized Ca where available) levels in the normal range in infants undergoing dialysis (level D, weak recommendation) 4.2.5 We recommend avoiding vitamin A supplementation in infants with CKD (level X, strong recommendation) 4.3 Phosphate binders and phosphate supplementation 4.3.1 Maintain P levels within the normal range for age, by adapting nutrition first but without compromising protein intake (level C, moderate recommendation) 4.3.2 Introduce P binders if serum P is not controlled with optimized nutritional management (level C, moderate recommendation) 4.3.3 We suggest using Ca-based P binders as first-line therapy (level D, weak recommendation) 4.3.4 We suggest considering sevelamer carbonate in infants with hypercalcemia (level D, weak recommendation) 4.3.5 We suggest providing P supplementation after optimization of nutritional phosphate intake in case of persistent hypophosphatemia (level D, weak recommendation) 4.4 Management of secondary hyperparathyroidism 4.4.1 We suggest starting active vitamin D in the lowest dose to achieve target PTH and normal calcium levels (level C, moderate recommendation) 4.4.2 We recommend giving native vitamin D and active vitamin D analogs directly by mouth, and avoiding nasogastric or gastrostomy tubes (level X, strong) 4.4.3 We recommend considering initiation or optimization of dialysis in infants with persistently uncontrolled SHPT and/or hyperphosphatemia despite an optimized nutritional and medical management (level C, moderate recommendation) 4.4.4 Cinacalcet may be considered with extreme caution in infants on dialysis who have persistent and severe hyperparathyroidism in the presence of high or high-normal calcium levels, despite optimized conventional management, including active vitamin D (level D, weak recommendation) 4.4.5 Parathyroidectomy may be considered as a last resort treatment when all medical management fails to control SHPT (level D, weak recommendation)

**Table 7:** Research questions

PICO question	Consensus statement
Clinical targets	To develop outcome and quality of life measures for neonates and young infants on kidney replacement therapy, similarly to what is done with the SONG (Standardized Outcomes in Nephrology) initiative in older children and adults
Biochemical targets	To determine the targets for the main biomarkers in this age group in case of CKD, and notably PTH, 25(OH)D, and ALP
Nutritional specificities	Balance studies to determine the true Ca requirements in infants for optimal bone quality and without a risk of vascular calcifications. To determine if urinary calcium excretion in healthy infants and those with CKD can be used to evaluate optimal calcium intakes
Therapeutic specificities	Cinacalcet data in infants in a real-life setting CKD-MBD in preterm infants

## Supplementary Information

Below is the link to the electronic supplementary material.

Graphical Abstract (PPTX 45 KB)

Supplementary file2 (DOCX 90 KB)
