# Supplementary material for: Diagnosis and management of mineral and bone disorders in infants with CKD: clinical practice points from the ESPN CKD-MBD and Dialysis working groups and the Pediatric Renal Nutrition Taskforce
Source: Pediatr Nephrol. 2023 Feb 14;38(9):3163–81. doi: 10.1007/s00467-022-05825-6 (PMC10432337; doi:10.1007/s00467-022-05825-6)
Supplement: Supplementary file 1 — Graphical Abstract (PPTX 45 KB) [file 467_2022_5825_MOESM1_ESM.pptx]

## Slide 1
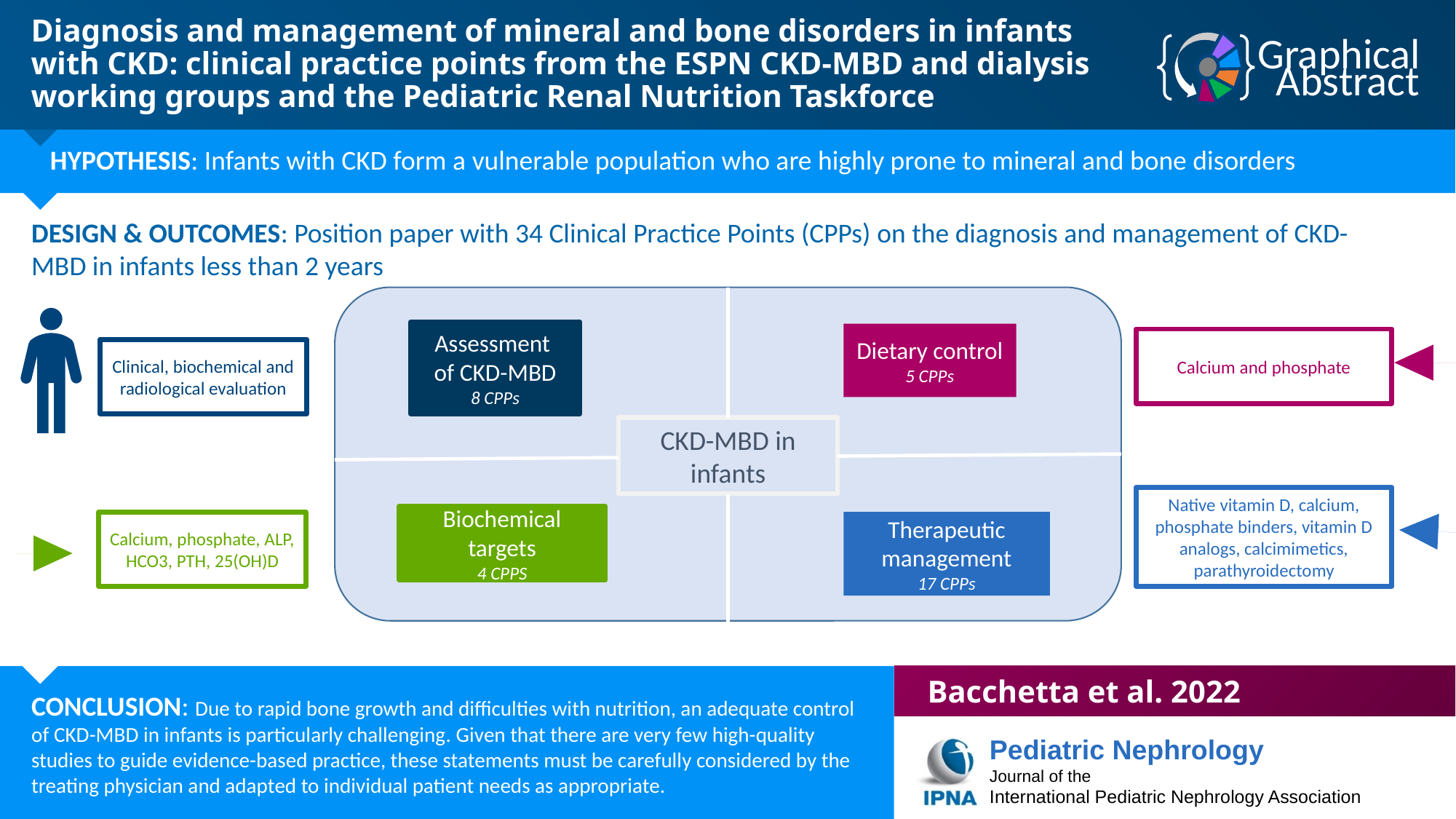

Diagnosis and management of mineral and bone disorders in infants with CKD: clinical practice points from the ESPN CKD-MBD and dialysis working groups and the Pediatric Renal Nutrition Taskforce
HYPOTHESIS: Infants with CKD form a vulnerable population who are highly prone to mineral and bone disorders
DESIGN & OUTCOMES: Position paper with 34 Clinical Practice Points (CPPs) on the diagnosis and management of CKD-MBD in infants less than 2 years
Assessment
of CKD-MBD
8 CPPs
Dietary control
5 CPPs
Calcium and phosphate
Clinical, biochemical and radiological evaluation
CKD-MBD in infants
Native vitamin D, calcium, phosphate binders, vitamin D analogs, calcimimetics, parathyroidectomy
Biochemical targets
4 CPPS
Calcium, phosphate, ALP, HCO3, PTH, 25(OH)D
Therapeutic management
17 CPPs
Bacchetta et al. 2022
CONCLUSION: Due to rapid bone growth and difficulties with nutrition, an adequate control of CKD-MBD in infants is particularly challenging. Given that there are very few high-quality studies to guide evidence-based practice, these statements must be carefully considered by the treating physician and adapted to individual patient needs as appropriate.
