# Supplementary material for: Diagnosis and management of mineral and bone disorders in infants with CKD: clinical practice points from the ESPN CKD-MBD and Dialysis working groups and the Pediatric Renal Nutrition Taskforce
Source: Pediatr Nephrol. 2023 Feb 14;38(9):3163–81. doi: 10.1007/s00467-022-05825-6 (PMC10432337; doi:10.1007/s00467-022-05825-6)
Supplement: Supplementary file 2 — Supplementary file2 (DOCX 90 KB) [file 467_2022_5825_MOESM2_ESM.docx]

**Supplemental Figure 1 Grading according to the American Academy of Pediatrics, adapted from** [38]**, and reproduced from** [39]

**Supplemental Table 1 Bone and musculo-skeletal clinical signs to search**

|  | **Clinical signs of bone impairment** |
| --- | --- |
| **Non-specific signs** | Bone deformities  Pain/discomfort at mobilization or movements  Fractures  Delayed motor milestones  Reduced height/length  Reduced height/length velocity and longitudinal growth |
| **Rickets** | Bowing and bending of the softened shafts of the long bones  Craniotabes, softness of the skull  Palpable enlargement of the chondro-costal junctions  Thickening of the wrists and/or ankles  Frontal prominence |
| **Secondary hyperparathyroidism** | Frontal prominence  Enlargement of the skull |

Delayed motor milestones and other disabilities may also be caused by other diseases, for example the primary genetic disease or the neurological consequences of extreme prematurity.

**Supplemental Table 2 Human milk and infant formulas most commonly used in UK: average calcium, phosphate and vitamin D content**

| **Per 100ml**  **Per standard dilution (~67kcal/100mL unless stated)** | **Calcium (mg/100 mL)** | **Phosphorous (mg/100 mL)** | **Vitamin D (IU/100 mL)** |
| --- | --- | --- | --- |
| **Mature human milk (69kcal/100mL)** | 34 | 15 | Trace |
| **First infant formulas (whey dominant)** | 51 | 32 | 59 |
| **Follow-on infant formulas (whey dominant)** | 69 | 47 | 68 |
| **Cow’s milk (63kcal/100mL)** | 120 | 96 | Trace |
| **Extensively hydrolysed formulas** | 68 | 43 | 67 |
| **Amino acid formulas** | 74 | 47 | 66 |
| **Renal specific infant formulas ( 100kcal/100mL)** | 35 | 21 | 96 |
| **Preterm/low birth weight formulas (80kcal/100mL)** | 110 | 71 | 130 |
| **Mature human milk with ~4-4.4g breast/human milk fortifier (85kcal/100mL)** | 105 | 56 | 180 |

*This table is for information only. It is purely to demonstrate the difference between calcium, phosphorus and Vitamin D content of various types of formula. The figures in the table should not be used in clinical practice. The composition of formulas in the UK as well as internationally can vary quite considerably. The authors strongly encourage readers to refer to the most up-to-date composition of their local formulas when making clinical decisions. The bioavailability of calcium from breast milk and infant formula is 66% and 40%, respectively, thus explaining the higher concentration of calcium in formulas.*
